# Skin Protective Effect of Epigallocatechin Gallate

**DOI:** 10.3390/ijms19010173

**Published:** 2018-01-06

**Authors:** Eunji Kim, Kyeonghwan Hwang, Jongsung Lee, Sang Yun Han, Eun-Mi Kim, Junseong Park, Jae Youl Cho

**Affiliations:** 1 Department of Genetic Engineering, Sungkyunkwan University, Suwon 16419, Korea; im144069@gmail.com (E.K); bioneer@skku.edu (J.L.); dangsukr@naver.com (S.Y.H); 2 Heritage Material Research Team, Amorepacific Research and Development Unit, Yongin 17074, Korea; khhwang@amorepacific.com (K.H.); emkim@amorepacific.com (E.-M.K)

**Keywords:** epigallocatechin gallate, skin hydration, antioxidant, anti-melanogenesis

## Abstract

Epigallocatechin gallate (EGCG) is a catechin and an abundant polyphenol in green tea. Although several papers have evaluated EGCG as a cosmetic constituent, the skin hydration effect of EGCG is poorly understood. We aimed to investigate the mechanism by which EGCG promotes skin hydration by measuring *hyaluronic acid synthase* (*HAS*) and *hyaluronidase* (*HYAL*) gene expression and antioxidant and anti-pigmentation properties using cell proliferation assay, Western blotting analysis, luciferase assay, 2,2-diphenyl-1-picrylhydrazyl (DPPH) assay, and reverse transcription polymerase chain reaction (RT-PCR) analysis. RT-PCR showed that EGCG increased the expression of natural moisturizing factor-related genes *filaggrin* (*FLG*), *transglutaminase-1*, *HAS-1*, and *HAS-2*. Under UVB irradiation conditions, the expression level of *HYAL* was decreased in HaCaT cells. Furthermore, we confirmed the antioxidant activity of EGCG and also showed a preventive effect against radical-evoked apoptosis by downregulation of caspase-8 and -3 in HaCaT cells. EGCG reduced melanin secretion and production in melanoma cells. Together, these results suggest that EGCG might be used as a cosmetic ingredient with positive effects on skin hydration, moisture retention, and wrinkle formation, in addition to radical scavenging activity and reduction of melanin generation.

## 1. Introduction

In our body, skin functions as a primary line of defense against external antigens and unwanted influences. Additional major functions of skin are feeling sensations and maintaining body temperature and moisture. Among these, the moisture retention effect is related to skin aging because it suppresses wrinkle formation. Skin aging occurs through two mechanisms—intrinsic and extrinsic aging. In intrinsic aging, decreased proliferative activities of skin cells lead to cellular senescence due to reduced synthesis of collagen and elastin. Extrinsic aging is caused by stimuli from the external environment such as ultraviolet (UV) irradiation, air pollution, and fine dust [1]. Skin aging induces dehydration of skin cells. The key molecule involved in skin hydration is hyaluronic acid (HA) [2,3]. HA has been reported to increase skin moisture by regulating the *hyaluronic acid synthase* (*HAS*) genes, and several studies have confirmed that retinoic acid (vitamin A) can effectively regulate HA in epidermis [4]. In the wound healing process, epidermal HA has been reported to promote cell proliferation and differentiation. The natural moisturizing factors (NMFs) are composed of HA and filaggrin (FLG), which directly or indirectly affect the skin moisture barrier [5]. However, there is little research on the mechanisms that regulate the NMFs. Several NMF components are susceptible to degradation and downregulation by UV irradiation. The mechanism by which UV induces skin damage is known to regulate signaling pathway components such as mitogen-activated protein kinases (MAPKs), nuclear factor (NF)-κB, and tumor necrosis factor (TNF)-α [6]. In addition, hyaluronidase (HYAL) is well known as an enzyme that hydrolyzes HA, and expression of the *hyaluronidase* (*HYAL*) gene is highly altered by ultraviolet (UV) irradiation [7].

The antioxidant defense system protects our skin from UV radiation, cigarette smoke, or hypoxia. Reactive oxygen species (ROS) induced by UV or oxidative stress enhance the progression of skin aging, skin wrinkling, and pigmentation [8,9]. Immoderate production of ROS activates internal cell programmed cell death, or apoptosis [10]. Apoptosis is considered to promote aging or age-associated diseases [11,12,13]. For these reasons, one approach to maintaining healthy skin or prevent aging is consumption of antioxidants to eliminate radicals.

Melanin is synthesized by transformation of l-tyrosine and protects our skin from external stress [14,15]. However, excessive melanin production can cause age spots or freckles. Many studies have reported the use of compounds that downregulate melanin synthesis as whitening constituents [15,16,17].

Epigallocatechin gallate (EGCG, (−)-epigallacatechin-3-gallate) is an abundant polyphenol derived from green tea (*Camellia sinesis* L. Ktze (Theaceae)). Green tea contains many catechins of which EGCG is the most abundant, accounting for more than 50% of the catechins, in addition to epicatechin-3-gallate (ECG), (−)-epigallocatechin (EGC), (−)-epicatechin (EC), and (+)-catechin [18,19]. EGCG has already been studied for many characteristics, such as antioxidant and anti-inflammatory activity and effects on proliferation, differentiation, and apoptosis [20,21]. However, the effect of EGCG on skin hydration has been marginally studied even though EGCG has been proposed as a potential ingredient for cosmetics [22,23]. The aim of this study is to investigate the mechanism by which EGCG promotes skin hydration through measurement of *HAS* and *HYAL* gene expression, antioxidant activity, and anti-pigmentation properties.

## 2. Results

### 2.1. Effect of EGCG on NMF Synthesis Activity

**Figure 1:**
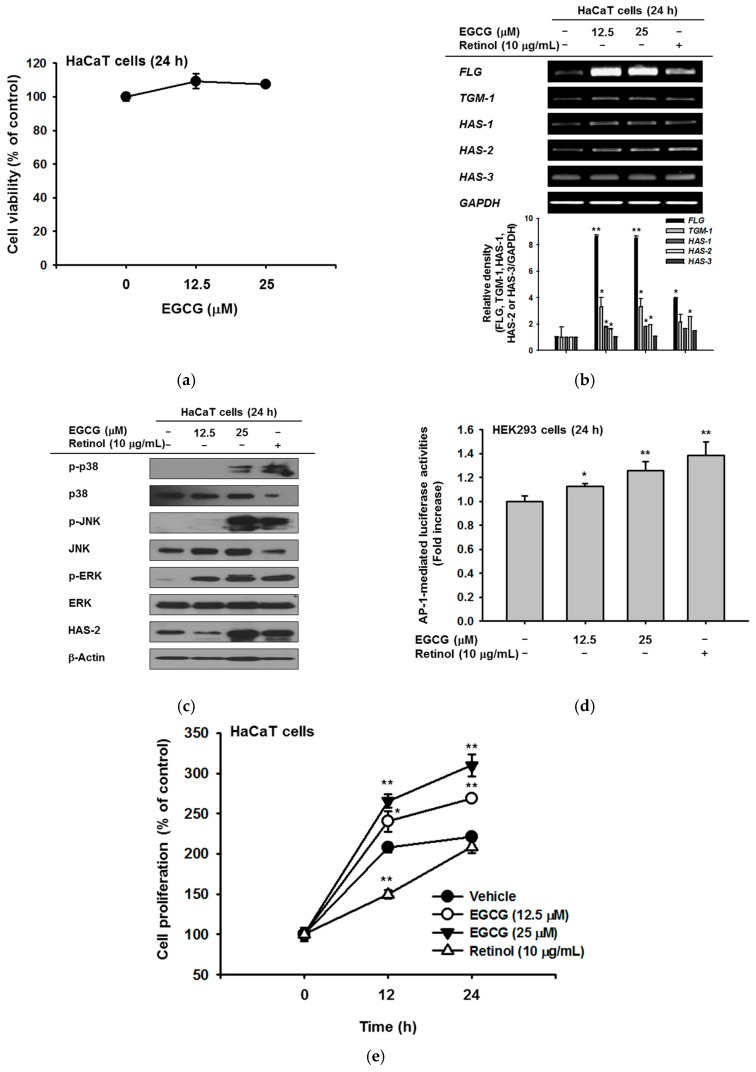
Effect of epigallocatechin gallate (EGCG) on skin hydration via natural moisturizing factors (NMF) synthesis. (**a**) the viability of EGCG–treated HaCaT cells was measured by 3-(4,5-dimethylthiazol-2-yl)-2,5-diphenyltetrazolium bromide (MTT) assay; (**b**) expression of NMF synthesis–related genes (*FLG*, *TGM*-*1*, and *HAS*-*1,2,3*) was measured by RT-PCR in HaCaT cells treated with EGCG (0–25 μM) or retinol (10 μg/mL) for 24 h; (**c**) the levels of mitogen-activated protein kinases (MAPKs) and HAS-2 proteins in cells treated with EGCG for 24 h were measured by immunoblot analysis; (**d**) HEK293 cells overexpressing activator protein (AP)-1-Luc were treated with EGCG for 24 h and luciferase activity was measured. β-galactosidase construct was used as a control; (**e**) proliferation of HaCaT cells was determined by MTT assay in cells treated with EGCG (0–25 μM) or retinol (10 μg/mL). Statistical significance of results ((**a**), bottom panel of (**b**,**d**,**e)**) was evaluated by Kruskal–Wallis/Mann–Whitney test. All data are expressed as means ± SD of an experiment. * *p* < 0.05 and ** *p* < 0.01 compared with control or normal groups.

EGCG was confirmed to show no cytotoxicity up to a concentration of 25 μM (Figure 1a). EGCG treatment increased the expression of genes related to NMF synthesis including *filaggrin* (*FLG*), *transglutaminase* (*TGM*)*-1*, and *hyaluronic acid synthase* (*HAS*)*-1*,*2*, and *3*. In particular, the level of *FLG* was significantly increased by EGCG compared with retinol treatment. The levels of *TGM-1*, *HAS-1*, and *HAS-2* were also augmented similar to the effect of retinol; the relative band density based on RT-PCR data significantly increased after EGCG treatment (Figure 1b). To investigate upstream proteins that regulate the NMF synthesis–related genes, we examined the protein expression of MAPKs and HAS-2. HAS-2 expression was increased by 25 μM EGCG. The expression of MAPKs, including c-Jun N-terminal kinase (JNK), extracellular-signal-regulated kinase (ERK), and p38, also increased with EGCG treatment (Figure 1c). To expand on the above results, we measured luciferase activity using an AP-1-Luc plasmid. AP-1-mediated luciferase activities were increased in a dose-dependent manner by EGCG treatment (Figure 1d). To examine whether EGCG promotes cell proliferation, HaCaT cells were treated with EGCG or retinol for 12 h and 24 h. EGCG enhanced cell proliferation to a greater extent than retinol. At 12 h, the percent increase in proliferation compared with 0 h was 240% for 12.5 μM EGCG (white circle) and 265% for 25 μM EGCG (black triangle), compared with only 150% for retinol (white triangle). At 24 h, the increase in proliferation was 269% for 12.5 μM EGCG, 310% for 25 μM EGCG, and 208% for retinol, compared with 0 h (Figure 1e).

### 2.2. Effect of EGCG on Skin Moisture Retention Activity

**Figure 2:**
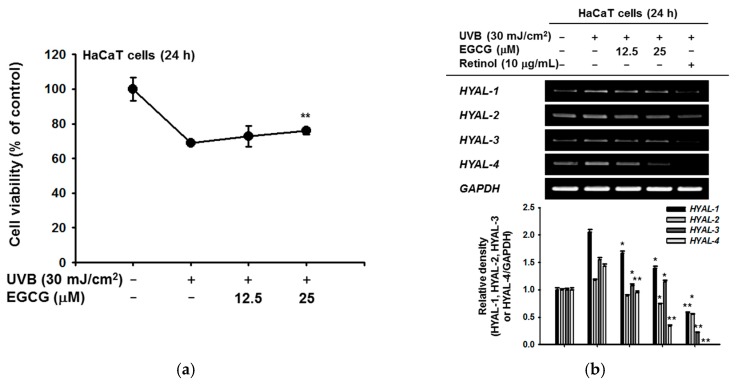
Effect of EGCG on skin hydration via inhibition of NMF degradation. (**a**) under UVB irradiation, the viability of HaCaT cells with and without EGCG was measured by MTT assay; (**b**) mRNA expression of *HYAL-1,2,3*, and *4* was determined by RT-PCR in HaCaT cells after irradiation with UVB (30 mJ/cm^2^) for 24 h and treatment with EGCG (0–25 μM) or retinol (10 μg/mL). The relative density of *HYAL* expression was also measured. Statistical significance of results ((**a**) and bottom panel of (**b**)) was evaluated by Kruskal–Wallis/Mann–Whitney test. All data are expressed as means ± SD of an experiment. * *p* < 0.05 and ** *p* < 0.01 compared with control group.

To investigate the skin protective activity against UVB irradiation, the cytotoxicity of EGCG against HaCaT cells was measured by the MTT assay. After exposure to UVB 30 mJ/cm^2^, the cell viability was decreased to 68.9% compared to the normal group. On the other hand, 12.5 μM EGCG increased the cell viability to 72.8%, and 25 μM EGCG significantly increased viability to 75.9%. EGCG therefore reduced the UVB-induced cell damage (Figure 2a). To confirm the moisture retention ability of HaCaT cells, we induced cell damage with UVB and measured *HYAL* gene expression by RT-PCR. The expression of *HYAL-2*, *-3*, and *-4* was decreased in the EGCG-treated group. In particular, under UVB-induced conditions, EGCG reduced the expression of *HYAL-4* in a dose-dependent manner (Figure 2b).

### 2.3. Antioxidant and Anti-Apoptotic Effect of EGCG

**Figure 3:**
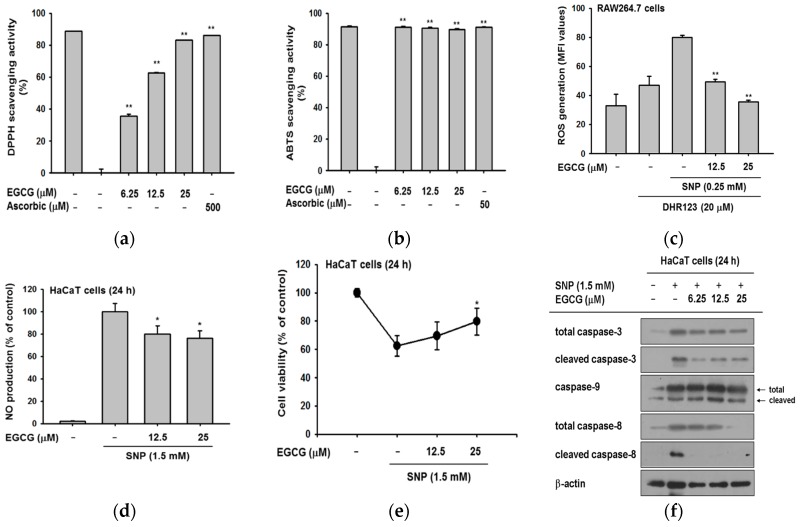
The antioxidative activities of EGCG. (**a**) EGCG (0–25 μM) was reacted with 2,2-diphenyl-1-picrylhydrazyl (DPPH) in the dark at 37 °C for 30 min. Absorbance at 517 nm was measured by spectrophotometry; (**b**) 2,2′-Azino-bis(3-ethylbenzothiazoline-6-sulphonic acid) (ABTS) and EGCG (0–25 μM) were incubated together at 37 °C for 30 min. Absorbance at 730 nm was measured by spectrophotometry. Ascorbic acid was used as a control compound; (**c**) RAW264.7 cells (1 × 10^6^ cells/well) were treated with dihydrorhodamine 123 (DHR123) (20 μM) in the dark at 37 °C for 10 min. Cells were treated with EGCG (0–25 μM) for 20 min, and incubated additionally with sodium nitroprusside (SNP). The ROS level was determined by flowcytometric analysis; (**d**) HaCaT cells were pretreated with EGCG and then exposed to SNP for 24 h. SNP-derived NO production was analyzed by Griess assay; (**e**) the viability of SNP-treated cells was determined by an MTT assay; (**f**) HaCaT cells were incubated with EGCG and SNP for 24 h. Cleaved and total forms of caspases and β-actin expression were determined by immunoblotting. Statistical significance of results (**a**–**e**) was evaluated by Kruskal–Wallis/Mann–Whitney test. All data are expressed as means ± SD of an experiment. * *p* < 0.05 and ** *p* < 0.01 compared with control group.

There are reports that EGCG has antioxidant activity by scavenging radicals [24,25], and it has been used as a positive control in several studies [26,27]. We confirmed the antioxidant effect of EGCG at target concentration (0–25 μM) by 2,2-diphenyl-1-picrylhydrazyl (DPPH) and 2,2′-Azino-bis(3-ethylbenzothiazoline-6-sulphonic acid) (ABTS) assays. EGCG significantly reduced DPPH radicals with a half maximal inhibitory concentration (IC_50_) value of 13.04 ± 3.95 μM (Figure 3a). In the ABTS assay (Figure 3b), EGCG clearly scavenged ABTS and its IC_50_ value was calculated as 1.57 ± 0.06 μM.

Based on these data, we examined the radical scavenging ability of EGCG against ROS induced by sodium nitroprusside (SNP). RAW264.7 cells were pretreated with EGCG and then with SNP and dihydrorhodamine 123 (DHR123) to test whether EGCG could regulate the intracellular ROS level. Measurement of the intracellular ROS by flowcytometric analysis showed that EGCG decreased the ROS level in a dose-dependent manner (Figure 3c). This indicated that EGCG could regulate not only extracellular but also intracellular radicals. Next, we examined whether SNP-induced nitric oxide (NO) production was deceased by EGCG. EGCG quenched SNP-induced NO production in HaCaT cells (Figure 3d). The cell viability of SNP-treated HaCaT cells was verified simultaneously since free radicals and ROS resulted in apoptosis. EGCG recovered the SNP-induced cell death, increasing cell viability to 80% (Figure 3e). These data showed that EGCG-treated cells were protected from oxidative conditions and SNP-induced cell death.

Antioxidants are widely known to suppress apoptosis by controlling ROS [10,28]. Based on the finding that EGCG could work as an antioxidant, we next explored the expression levels of apoptotic molecules to determine how EGCG protects from cell death. In apoptosis, caspases are cleaved and turned into active forms [29]. The amount of cleaved caspase-3 was dramatically reduced by EGCG (Figure 3f). Since caspase-3 is the common effector molecule of extrinsic and intrinsic apoptotic pathway, we examined which apoptotic pathway was controlled by EGCG [30,31]. Evaluation of caspase-9 and -8 by immunoblotting showed that EGCG exclusively suppressed the formation of cleaved caspase-8. These results implied that EGCG prevents apoptosis by inhibiting the extrinsic apoptotic pathway.

### 2.4. Antioxidant and Anti-Apoptotic Effect of EGCG

**Figure 4:**
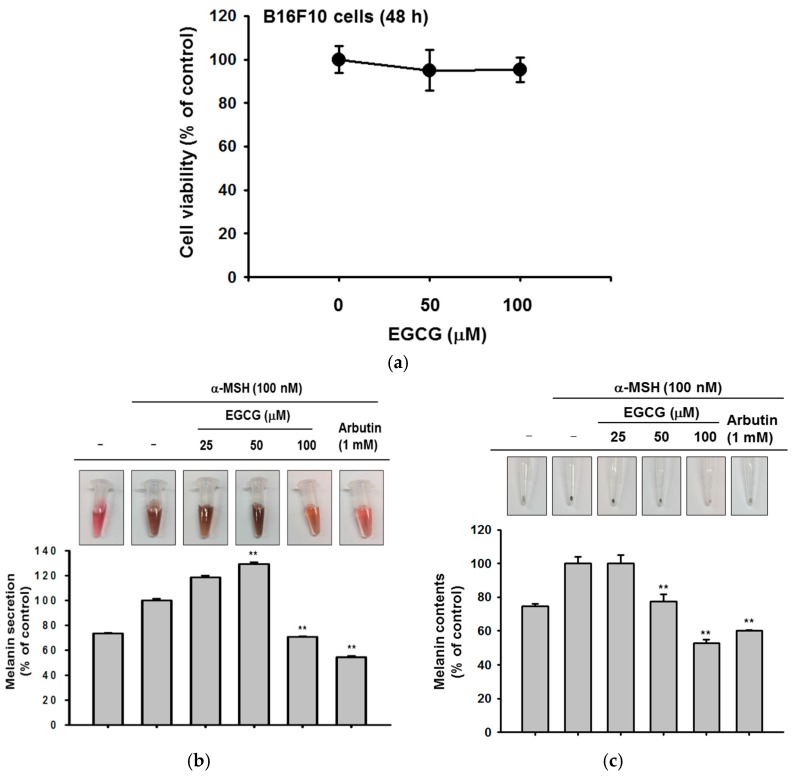
The effect of EGCG on melanin secretion and generation. (**a**) B16F10 cells were treated with EGCG (0–100 μM) for 24 h. The toxicity of EGCG was measured by MTT assay; (**b**,**c**) B16F10 cells were incubated with EGCG and α-melanocyte-stimulating hormone (α-MSH) for 48 h. Cell culture media were collected for measurement of extracellular melanin secretion, and cell lysates were used to verify melanin generation. Absorbance of supernatants was measured at 475 nm. The pellets of cells were lysed, and the absorbance of B16F10 cell lysates was measured at 405 nm. Statistical significance of results ((**a**), and bottoms of (**b**,**c**)) was evaluated by Kruskal–Wallis/Mann–Whitney test. All data are expressed as means ± SD of an experiment. * *p* < 0.05 and ** *p* < 0.01 compared with control group.

Last, we determined whether EGCG could regulate melanin secretion and generation in B16F10 cells. Melanin pigmentation was induced by α-melanocyte stimulating hormone (αMSH), and arbutin was used as a positive control compound. EGCG significantly reduced extracellular melanin secretion at 100 μM without cell cytotoxicity (Figure 4a,b). Simultaneously, we measured the melanin content in cells. Although EGCG did not affect melanin secretion at 50 μM, the production of melanin was decreased by a concentration of 50 μM or higher (Figure 4c). These results showed that EGCG regulates melanin pigmentation and might be used as a whitening ingredient in cosmetics.

## 3. Discussion

Moisturizing ability is one of the most basic and important features of cosmetic ingredients and it is therefore necessary to study mechanisms of controlling the moisturizing power. Many previous papers have used HAS as a marker to measure moisture. As animal experiments have recently been banned in the cosmetic field and the transepidermal water loss measuring method was also unavailable, we tried to find an alternative way to measure moisture in vitro. Our study suggests alternative testing methods using RT-PCR and Western blot. In the present study, we analyzed whether EGCG can improve the moisturizing power in keratinocytes. We first confirmed the mRNA expression of NMF-related genes (*FLG*, *TGM1*, *HAS-1*, *-2*, and *-3*). EGCG increased the expression of all NMF-related genes without cytotoxicity (Figure 1a,b). Our results suggested that EGCG upregulates *FLG*, *TGM1*, *HAS-1*, and *HAS-2* in keratinocytes, thereby providing moisture to the epidermis layer, which can maintain the skin barrier more firmly. To identify proteins that regulate NMF, the levels of MAPKs and HAS-2 protein were measured by immunoblotting. We found that EGCG increased the phosphorylation of p38, ERK, and JNK to the same extent as retinol (a moisturizing positive control), and HAS-2 was also upregulated by treatment with EGCG at 25 μM (Figure 1c). These results demonstrated that NMF-related genes are regulated through MAPKs. Activity of AP-1, the transcription factor of MAPKs, was examined by the luciferase system. EGCG dose-dependently enhanced AP-1-mediated luciferase activity (Figure 1d). Therefore, we confirmed that MAPKs regulate keratinocyte moisturizing. As previous papers used UV irradiation [7,32], we examined whether EGCG affects moisture levels in UV irradiation conditions. Under UV irradiation, EGCG reduced cellular damage (Figure 2a) and the expression levels of HYALs (Figure 2b). EGCG inhibited the degradation of HA in the epidermis by reducing the level of HYAL expression, and increased the hydration retention capacity of the skin barrier.

In some papers using keratinocytes, the skin barrier was filled with keratinocytes and wrinkle formation was reduced through cell proliferation [33]. Thus, HaCaT cells were treated with EGCG, and cell proliferation was observed up to 24 h. Our data showed that EGCG increased cell proliferation (Figure 1e). Interestingly, EGCG was found to be beneficial not only for miniaturization, but also as an anti-wrinkle agent.

When skin is exposed to UV, oxidative stress, or environmental pollutants, free radical and ROS levels are elevated and could cause skin damage. Constant exposure to free radicals or ROS may promote the development of inflammatory skin diseases, vitiligo, or skin cancer [34,35]. For these reasons, elimination of free radicals and ROS by antioxidants is critical to protect our skin from harmful factors and maintain a healthy skin condition. Here, we confirmed that EGCG possessed antioxidant capacity, and consequently protected keratinocytes from SNP- and UV-mediated radicals (Figure 3). The anti-apoptotic effect arising from antioxidant activity was also evaluated. Free radicals are considered to be key molecules accelerating skin aging [13]. Together, our data suggest that EGCG might be used as a cosmetics ingredient with antioxidant and skin protective effects.

There are a number of reports regarding the antioxidant ability of EGCG [36,37,38]. Based on its activity as a powerful antioxidant, numerous researchers have suggested that EGCG could be used as an anti-HIV agent, anti-inflammatory compound, or protector from DNA damage [39,40]. Furthermore, to improve the antioxidant property, EGCG derivatives were developed and exhibited greater antioxidant ability [41]. EGCG is expected to be further developed in several directions and shows promise for applications in cosmetics. In terms of this, animal experiments with EGCG should be avoided. Since this could be a big limitation to understand exact action mechanism of EGCG in in vivo conditions, alternative experimental approaches with artificial skin conditions are needed.

Although in vitro activity of EGCG regarding skin protection is promising, the effect of this compound in real human skin should be tested. In fact, several previous papers including animal experiments have proposed a possibility that EGCG could be effective in human skin. Thus, it was found that topically administered EGCG exerted the inhibition of photocarcinogenesis in BALB/cAnNHsd mice under UV treatment conditions [42]. UVB exposure-induced immunosuppressive events such as reduction of the number of CD11b^+^ monocytes/macrophages and decrease in neutrophils infiltrating into skin inflammatory lesions were also recovered by topical application of EGCG [43]. Moreover, UVB-induced erythema in hairless mice was also significantly reduced in the EGCG treated group [44]. Topical treatment of EGCG was found to downregulate UVB-induced oxidative stress such as lipid peroxidation and protein oxidation. These data strongly implicated that EGCG could be effective in in vivo conditions during UV irradiation via increasing anti-oxidative activity. In spite of several animal experiments, unfortunately, scientific evidence observed in human studies with EGCG has not been sufficiently accumulated. Therefore, we are now planned to implement clinical trial to prove the possibility using several parameters such as levels of skin moisturizing factor genes, antioxidant activity, and melatonin amount.

**Figure 5:**
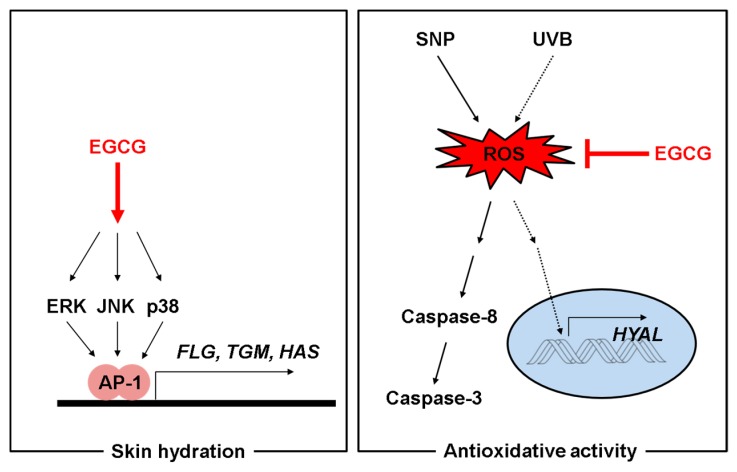
Mechanisms of skin hydrating and antioxidant activities of EGCG. EGCG upregulated expression of skin hydrating genes in HaCaT cells. EGCG possessed antioxidative ability and cleared the radicals from SNP and UVB. It also inhibited activation of caspases-8 and -3, and expression of HYALs. Red arrow: Activation pathway by EGCG, and red line: Inhibition pathway by EGCG.

In summary, our results suggest that EGCG could be employed as an effective cosmetics ingredient with skin moisturizing, antioxidant, and anti-melanogenesis activity, as summarized in Figure 5. For ensuring its protective activity in human skin, additional clinical studies with healthy volunteers will be continued in the following studies.

## 4. Materials and Methods

### 4.1. Materials

Cell lines (HaCaT, HEK293, and B16F10) were purchased from American Type Culture Collection (Rockville, MD, USA). Dulbecco’s modified Eagle’s medium (DMEM), RPMI1640, fetal bovine serum (FBS), phosphate buffered saline (PBS), and penicillin-streptomycin were purchased from HyClone (Logan, UT, USA). 3-(4-5-Dimethylthiazol-2-yl)-2,5-diphenyltetrazolium bromide (MTT) was purchased from Amresco (Brisbane, Australia). Polyethylenimine (PEI), 1-diphenyl-2-picryl-hydrazyl (DPPH), 2,2′-azino-bis(3-ethylbenzothiazoline-6-sulphonic acid) diammonium salt (ABTS), ascorbic acid, sodium nitroprusside (SNP), dehydrorhodamine 123 (DHR123), α-melanocyte stimulating hormone (αMSH), TRIzol, and arbutin were purchased from Sigma Aldrich Chemical Co. (St. Louis, MO, USA). The cDNA synthesis kit was purchased from Thermo Fisher Scientific (Waltham, MA, USA). Forward and reverse primers for polymerase chain reaction (PCR) were synthesized by Macrogen (Seoul, Korea) and PCR premix was purchased from Bio-D Inc. (Seoul, Korea). The luciferase assay system was purchased from Promega (Madison, WI, USA). Polyvinylidenedifluoride (PVDF) membrane was from Merck Millipore (Billerica, MA, USA). Antibodies against cleaved or total forms of caspase-3, caspase-8 and caspase-9, and phospho- or total forms of p38, ERK, JNK, and β-actin were obtained from Cell Signaling Technology (Beverly, MA, USA). Anti-HAS-2 antibody was purchased from Santa Cruz Biotechnology (Santa Cruz, CA, USA).

### 4.2. Cell Culture

HaCaT cells and B16F10 cells were cultured in DMEM with 10% FBS and 1% penicillin-streptomycin at 37 °C in a 5% humidified incubator. RAW264.7 cells were cultured in RPMI 1640 with 10% FBS and 1% penicillin-streptomycin. HEK293 cells were incubated in DMEM supplemented with 5% FBS and 1% penicillin-streptomycin.

### 4.3. Cell Viability and Cell Proliferation Assay

HaCaT cells or B16F10 cells were seeded at 3 × 10^4^ cells per well in 96-well plates for 24 h and then treated with EGCG for 24 h. Cell viability was measured using MTT assay as reported previously [45]. Cells were incubated with 10 μL/well MTT solution for 3–4 h and then 100 μL of MTT stop solution (10% sodium dodecyl sulfate containing 1M HCl) was added. After 8 h, solubilized formazan was measured by absorbance at 570 nm using an optical density reader (BioTek, Winooski, VT, USA). HaCaT cells were seeded at 3 × 10^3^ cells per well in 96-well plates and then treated with EGCG (12.5 and 25 μM) for 12 h and 24 h. After incubation for 12 h and 24 h, the MTT assay was performed as above.

### 4.4. RT-PCR Analysis

All mRNA analyses using HaCaT cells were performed in 6-well plates. Total mRNA was precipitated using TRIzol reagent according to the manufacturer’s instructions. The concentration of mRNA was measured by spectrophotometry for complementary DNA (cDNA) synthesis. cDNA was synthesized using a cDNA synthesis kit. Primers for genes were designed on website (Available online: https://www.ncbi.nlm.nih.gov/tools/primer-blast/). Reverse transcription polymerase chain reaction was conducted using specific forward and reverse primers as reported previously [46]. Primers are listed in Table 1.

### 4.5. DPPH Assay

The scavenging effect of EGCG was determined by a DPPH decoloration assay [47]. DPPH was dissolved in methanol at a final concentration of 250 mM. A mixture of 475 mL DPPH and 5 mL EGCG (0–25 μM) or ascorbic acid (500 μM) was incubated at 37 °C for 30 min. Absorbance at 517 nm of each fraction was measured by spectrophotometry. The DPPH scavenging effect was expressed as a percentage:

DPPH scavenging effect (%) = [(A0 − A1)/A0] × 100(1)

where A0 is the absorbance of DPPH and A1 is the absorbance of samples.

### 4.6. ABTS Assay

ABTS assay was performed as reported previously [48]. Briefly, 7.4 mM ABTS solution and 2.4 mM potassium persulfate solution were mixed at 1:1 ratio and incubated at room temperature overnight to generate ABTS radical cation (ABTS•^+^). ABTS solutions were transferred to 96-well plates and EGCG (0–25 μM) or ascorbic acid (50 μM) was added to each well. Mixtures were incubated at 37 °C for 30 min. The absorbance at 730 nm was measured. ABTS scavenging effect was expressed as a percentage:

ABTS scavenging effect (%) = [(A0 − A1)/A0] × 100(2)

where A0 is the absorbance of ABTS and A1 is the absorbance of samples.

### 4.7. ROS Generation Assay

The level of intracellular ROS was determined by recording the change in fluorescence that resulted from oxidation of the fluorescent probe DHR123. Briefly, 1 × 10^6^ RAW264.7 cells were exposed to EGCG for 30 min and then incubated with SNP (0.25 mM) at 37 °C for 20 min to induce ROS production. The cells were additionally incubated with 20 μM of the fluorescent probe DHR123 for 30 min at 37 °C. The degree of fluorescence, which corresponded to the level of intracellular ROS, was determined using a FACScan flow cytometer (Becton-Dickinson, San Jose, CA, USA) as reported previously [49].

### 4.8. Preparation of Cell Lysates and Immunoblotting Analysis

HaCaT cells (4 × 10^5^ cells/mL) were washed three times with cold PBS with 1 mM sodium orthovanadate and resuspended in lysis buffer (20 mM Tris-HCl, pH 7.4, 2 mM ethylenediaminetetraacetic acid (EDTA), 2 mM ethyleneglycoltetraacetic acid, 50 mM β-glycerophosphate, 1 mM orthovanadate, 1 mM dithiothreitol, 1% Triton X-100, 10% glycerol, 10 μg/mL aprotinin, 10 μg/mL pepstatin, 1 mM benzamidine, and 2 mM phenylmethylsulfonyl fluoride (PMSF)). The lysates were clarified by centrifugation at 12,000 rpm for 10 min at 4 °C and stored at −20 °C until use. The protein content of the supernatants was measured using the Bradford assay [50]. The soluble fractions of the cell lysates were immunoblotted, and the phospho- or total levels of caspase-3, caspase-9, caspase-8, p38, JNK, ERK, HAS-2, and β-actin were visualized as previously reported [51].

### 4.9. Luciferase Reporter Gene Assay

HEK293 cells were seeded at 1.2 × 10^4^ cells per well in 24-well plates. After 24 h, cells were transfected with β-galactosidase and AP-1-Luc for 24 h. The transfection reagent used was PEI as reported previously [52]. The cells were treated with EGCG for 24 h. Luciferase assay was conducted using the Luciferase Assay System.

### 4.10. UVB Irradiation

HaCaT cells were seeded at 7 × 10^5^ cells per well in 6-well plates and incubated under starvation conditions for 24 h using serum-free MEM. HaCaT cells were pretreated with EGCG for 30 min, washed with PBS, and exposed to UVB irradiation (UVB lamp: Bio-link crosslinker BLX-312; Vilber Lourmat, Collegien, France) at a dose of 30 mJ/cm^2^. After UVB irradiation, DMEM medium containing EGCG was added and the cells were incubated for 24 h.

### 4.11. Measurement of Melanin Secretion and Contents

B16F10 cells (1 × 10^5^ cells/mL) were co-treated with αMSH and EGCG (0–100 μM) or 1 mM arbutin for 48 h. Cell culture media were transferred for measurement of melanin secretion Absorbance of the media was measured at 475 nm. Cells were washed with cold PBS and harvested. For measurement of melanin content, cells were lysed with 20 mL cell lysis buffer (50 mM Tris-HCl pH 7.5, 20 mM NaF, 25 mM β-glycerolphosphate pH 7.5, 120 mM NaCl and 2% NP-40 in distilled water) and centrifuged at 12,000 rpm for 10 min. The supernatants were removed and the pellet was dissolved in 100 mL 1 M NaOH containing 10% DMSO at 60 °C for 30 min. The absorbance of each fraction was measured at 405 nm [53].

### 4.12. Statistical Analysis

All data of this study are expressed as means ± standard deviations (SDs) of an experiment performed with six (Figure 1a,d,e, Figure 2a, Figure 3c–e and Figure 4a), three (Figure 3a,b and Figure 4b,c) or two (Figure 1b bottom panel and Figure 2b bottom panel) technical replicates per group. For statistical comparison, results were analyzed by ANOVA with Scheffe’s post hoc test, Kruskal-Wallis and Mann-Whitney *U* tests. For all analyses, *p* < 0.05 was considered statistically significant. All statistical tests were performed with SPSS software (SPSS Inc., Chicago, IL, USA). Similar experimental data were also observed using an additional independent set of experiments that was conducted using the same numbers of samples.

## Figures and Tables

**Table 1:** Primers used for reverse transcriptase polymerase chain reaction.

Name		Sequence (5′ to 3′)
*FLG*	F	AGGGAAGATCCAAGAGCCCA
	R	ACTCTGGATCCCCTACGCTT
*TGM*	F	GAAATGCGGCAGATGACGAC
	R	AACTCCCCAGCGTCTGATTG
*HAS-1*	F	CCACCCAGTACAGCGTCAAC
	R	CATGGTGCTTCTGTCGCTCT
*HAS-2*	F	TTCTTTATGTGACTCATCTGTCTCACCGG
	R	ATTGTTGGCTACCAGTTTATCCAAACG
*HAS-3*	F	TATACCGCGCGCTCCAA
	R	GCCACTCCCGGAAGTAAGACT
*HYAL-1*	F	CAGAATGCCAGCCTGATTGC
	R	CCGGTGTAGTTGGGGCTTAG
*HYAL-2*	F	TACACCACAAGCACGGAGAC
	R	ATGCAGGAAGGTACTGGCAC
*HYAL-3*	F	CCAGGATGACCTTGTGCAGT
	R	CCATCTGTCCTGGATCTCGC
*HYAL-4*	F	TGAGCTCTCTTGGCTCTGGA
	R	AGGCAGCACTTTCTCCTATGG
*GAPDH*	F	GCACCGTCAAGGCTGAGAAC
	R	ATGGTGGTGAAGACGCCAGT

F: Forward; R: Reverse.
